# Dynamic and Static Stabilization of Anterior Shoulder Instability With the Subscapular Sling Procedure

**DOI:** 10.1016/j.eats.2021.03.027

**Published:** 2021-06-20

**Authors:** Jan Arild Klungsøyr, Terje Vagstad, Peter Johannes Klungsøyr, Alf Inge Hellevik, Jon Olav Drogset

**Affiliations:** a Norwegian University of Science and Technology (NTNU), Faculty of Medicine and Health Sciences, Trondheim, Norway; b Department of Orthopaedic Surgery, Ålesund Hospital, Møre and Romsdal Hospital Trust, Ålesund, Norway; c Department of Orthopedic Surgery, Trondheim University Hospital and Norwegian University of Science and Technology (NTNU), Trondheim, Norway

## Abstract

There are numerous arthroscopic techniques available to address anterior shoulder instability. Complications are various, and in pursuit of new treatment options, an alternative arthroscopic technique with less potential for complications has been developed. The novel subscapular sling with a semitendinosus graft provides both dynamic and static stability. This procedure uses a semitendinosus graft as a sling around the upper two-thirds of the subscapular tendon, attached to the anterior glenoid rim. The sling phenomenon present in the Latarjet procedure was the basis of the development. The efficacy of the subscapular sling procedure has been verified in biomechanical studies and further investigated in a clinical pilot study. The procedure can be performed without altering the anatomy of nearby structures such as the coracoid process, the conjoined tendon, and the axillary and musculocutaneous nerves. The authors propose the arthroscopic subscapular sling procedure as an alternative to existing surgical treatment options for recurrent anterior shoulder instability.

Anterior shoulder instability is a challenge both for patients and surgeons. There are many treatment options available, depending on the structures injured.^1^ In cases of mere anterior soft-tissue damage, the traditional Bankart repair may be used.^2^ Bone-transferring procedures are preferable in shoulders with severe glenoid bone loss and residual luxation following previous soft-tissue stabilization.^3^, ^4^, ^5^ Both Bankart and bone-grafting procedures been known to have complications.^6^, ^7^, ^8^, ^9^, ^10^, ^11^ The potential for better treatment options led to the development of the subscapular sling with a semitendinosus (ST) graft.^12^^,^^13^ Feasibility and biomechanical cadaveric studies have been performed,^12^^,^^13^ and a clinical pilot study of the subscapular sling shows promising preliminary results. The purpose of this paper is to present the surgical procedures involved in the subscapular sling method with an ST graft. The ST graft is pulled through a slit in the subscapular tendon and attached to the anterior glenoid rim to replace the torn labrum. A sling is constructed around the upper two-thirds of the subscapular tendon and provides, together with the reconstruction of the labrum, both static and dynamic stabilization of the shoulder (Fig 1).

**Fig 1:**
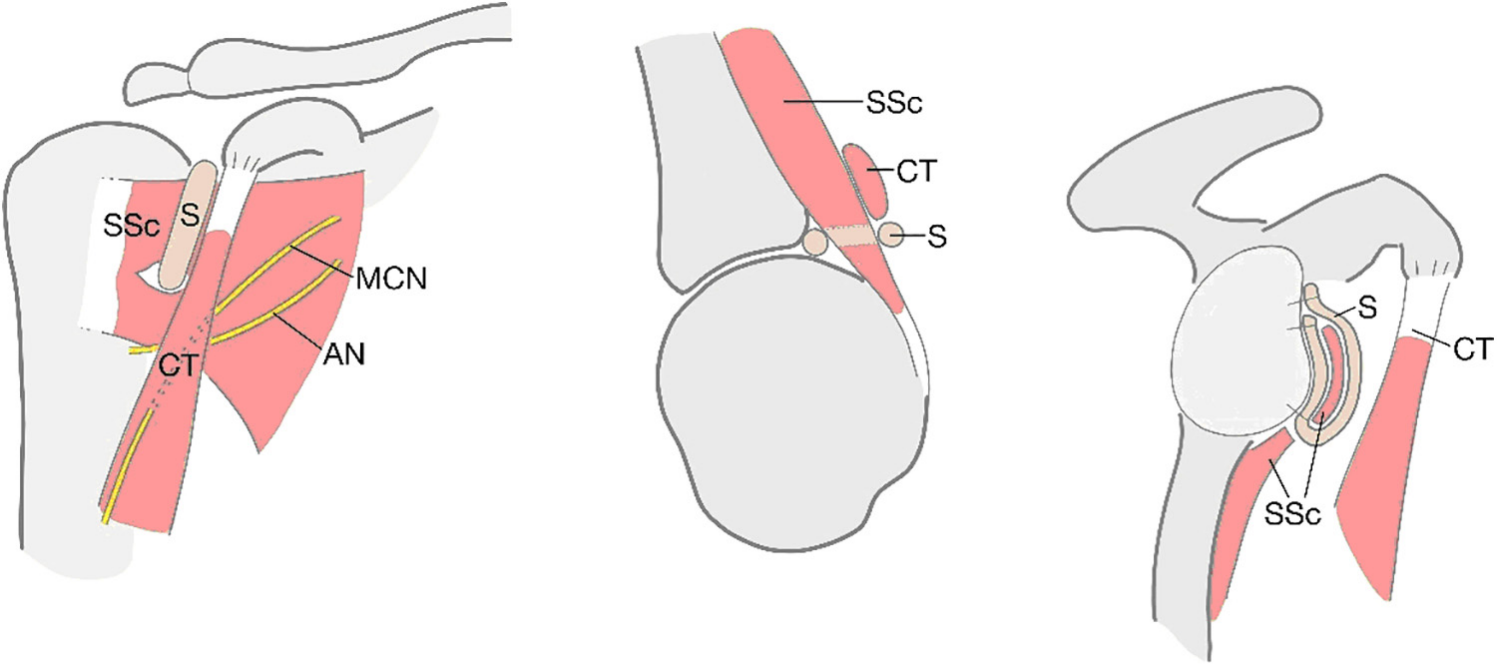
Schematic drawings of the subscapular sling. Sling (S), subscapular tendon (SSc), conjoined tendon (CT), axillary nerve (AN), and musculocutaneous nerve (MCN).

## Surgical Procedure (With Video Illustration)

**Fig 2:**
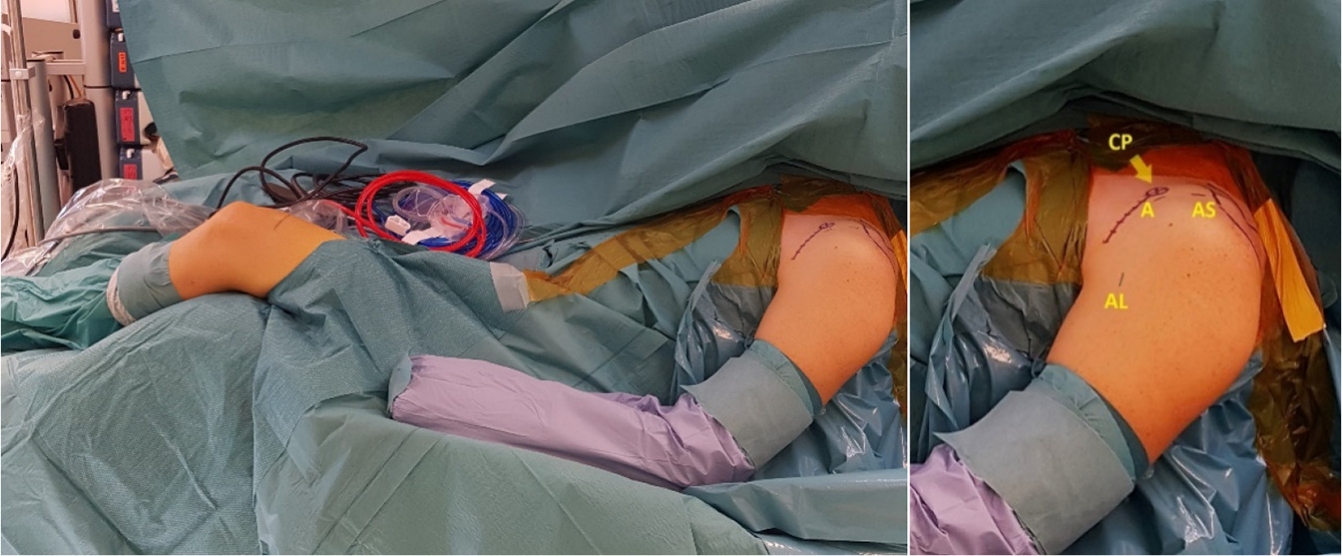
Patient in beach chair position during surgery of the left shoulder. Coracoid process (CP). Posterior portal for viewing; the anterosuperior (AS), anterior (A), and anterior lateral portal (AL) for instrumenting.

The surgical procedure is illustrated in Video 1. The patient is placed in the beach chair position under general anesthesia with an optional brachial plexus block. For ease of graft harvest and patient positioning, the ipsilateral knee is used when possible. The knee is draped, and local anesthesia with adrenalin is applied to avoid the use of a tourniquet. The anatomic landmarks of the shoulder are marked (Fig 2). Standard posterior portal, anterior, and anterosuperior portals are positioned with the anterior portal cranial to the superior edge of the subscapular tendon.

**Fig 3:**
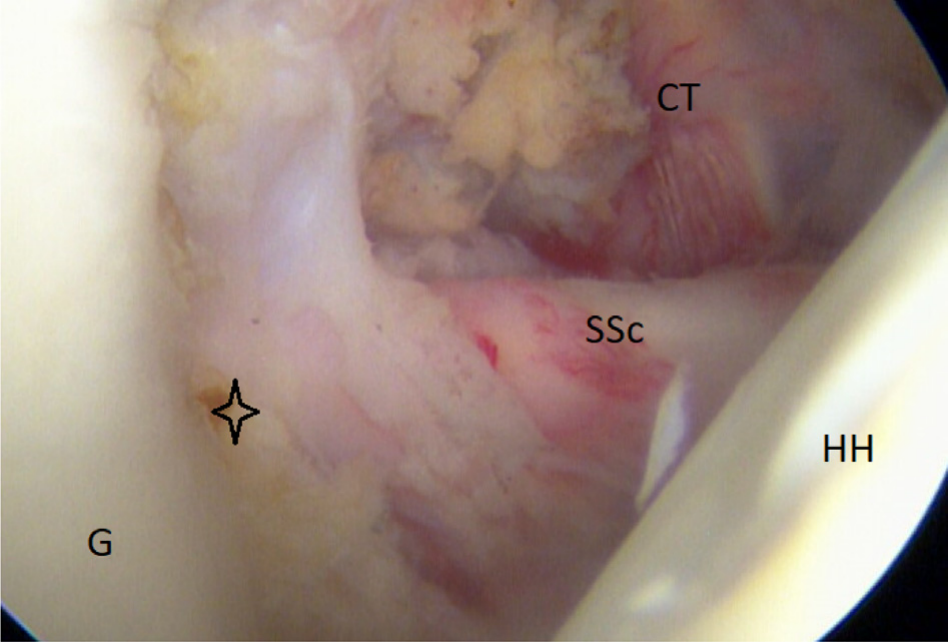
Arthroscopic view of a right shoulder, posterior viewing portal. The star sign indicates the lost anterior labrum on the glenoid (G). (CT conjoined tendon; HH, humeral head; SSc, subscapular tendon.)

A diagnostic arthroscopy is performed after an external examination of the shoulder stability. The rotator cuff and in particular the subscapular tendon are inspected to rule out injuries. Furthermore, the amount of glenoid bone loss, on/off-track Hill–Sachs lesions, and labral and ligament ears are assessed (Fig 3).

**Fig 4:**
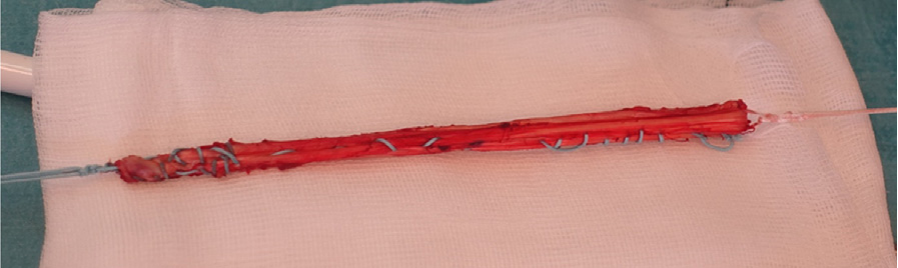
Semitendinosus graft doubled and whipstitched ready for fixation.

The ST tendon is identified through a small skin incision on the proximal medial tibia and harvested with a corkscrew tendon stripper. The graft is doubled, whip stitched, and the length is measured. Additional sutures are placed at both ends of the graft to ease the advancement into the joint and around the subscapular tendon before fixation (Fig 4). Before skin closure, a 12-gauge catheter is advanced on the medial side of the thigh along the remaining hamstrings tendon to administer ropivacaine.

**Fig 5:**
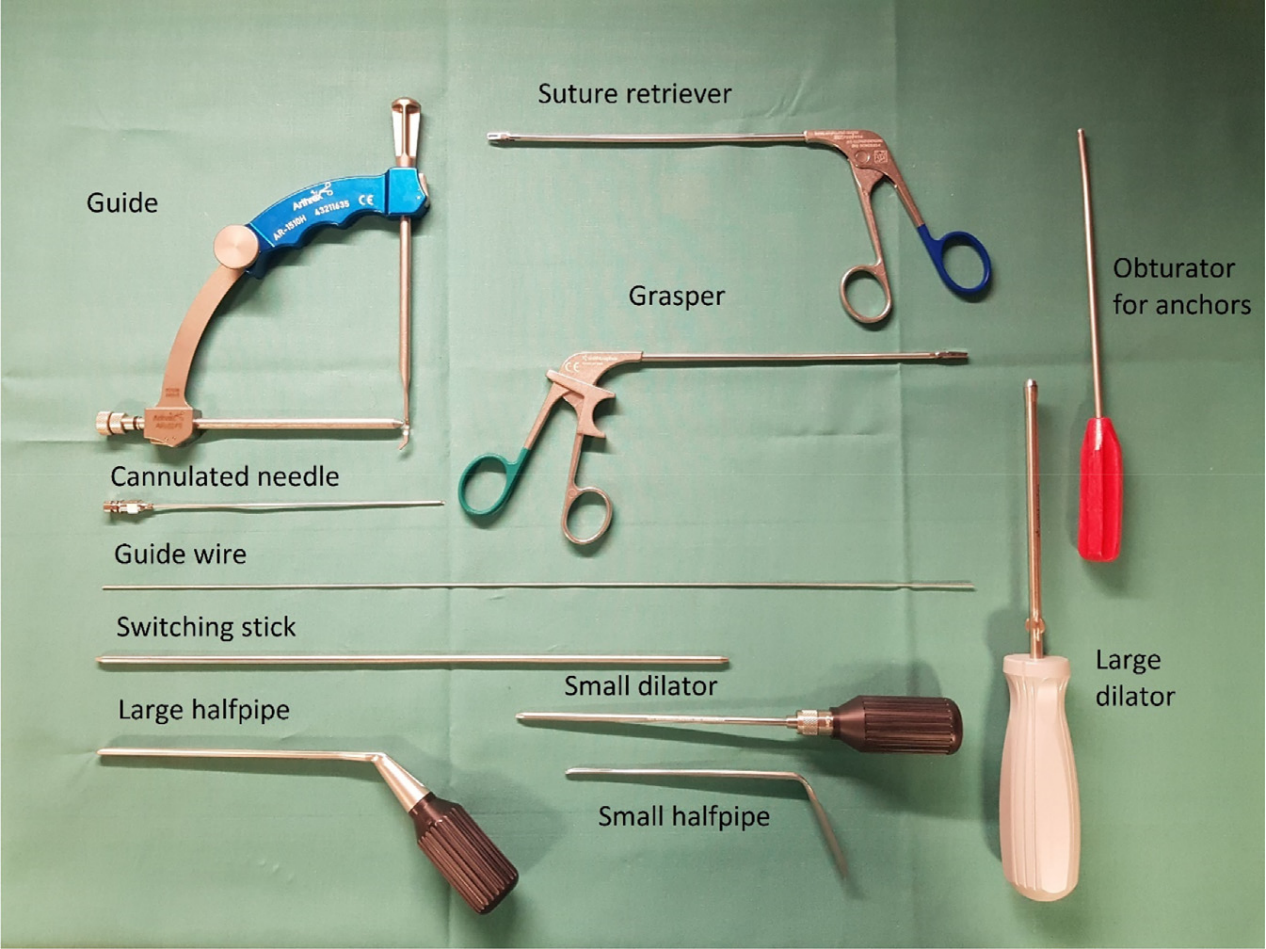
Arthroscopic instruments needed to perform the subscapular sling procedure.

**Fig 6:**
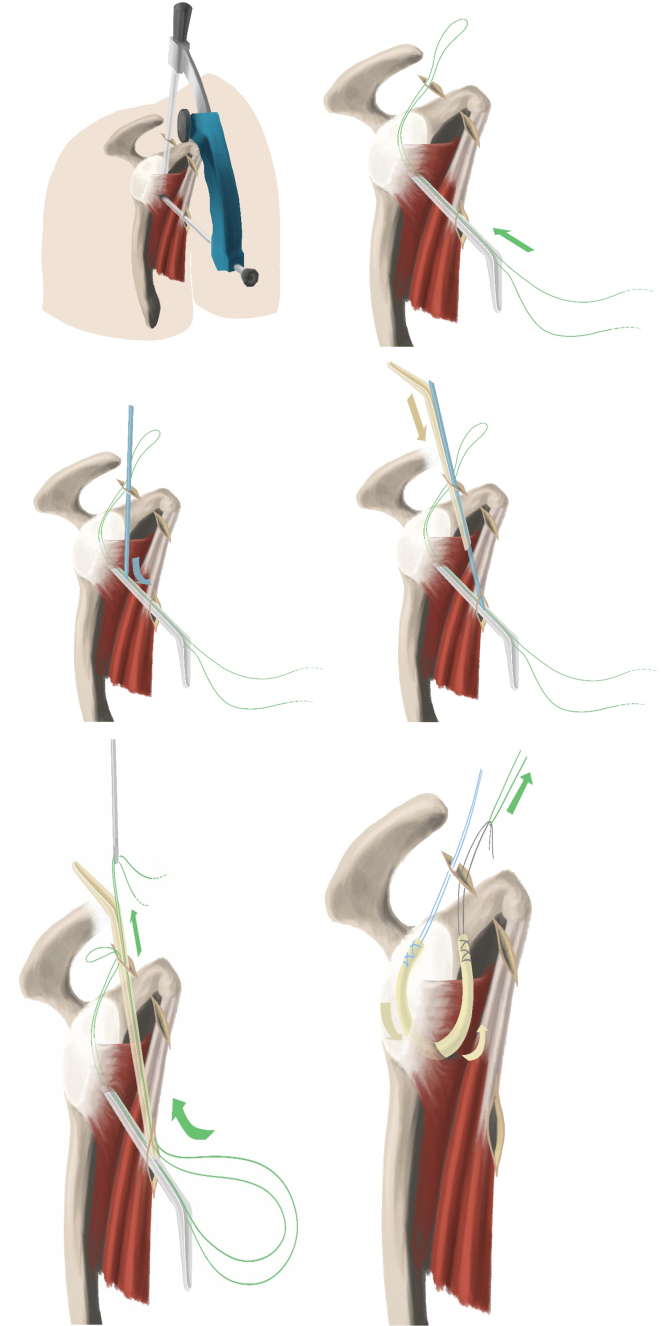
Schematic drawings of a right shoulder. The aiming guide and the creation of the slit in the subscapular tendon with halfpipes is demonstrated. The green pulling suture advances the semitendinosus graft into the glenohumeral joint and around the subscapular tendon.

**Fig 7:**
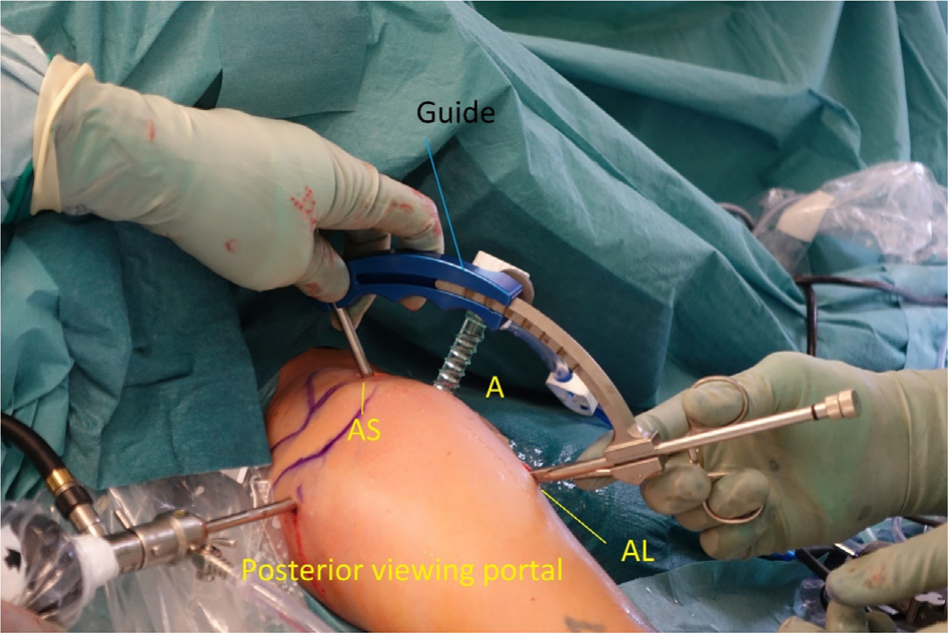
Right shoulder. The correct placement of the aiming guide in the anterosuperior (AS) and anterolateral (AL) portals. An 8-mm arthroscopic cannula is in the anterior portal (A).

Remnants of the anterior labrum are removed from the 2- to 5-o'clock position using different rasps, shaver, and radiofrequency coblation. The anterior glenoid must be cleared of soft tissue to support ingrowth of the ST graft. The anterior portion of the subscapular tendon is cleared, and the axillary nerve is identified and kept at a distance from the portals and instruments during surgery. All instrumentation is performed lateral to the conjoined tendon, thus also preserving the musculocutaneous nerve, which is not visualized. The lower anterolateral portal is created lateral to the conjoined tendon from anterior to posterior direction using an aiming guide, cannulated needle, guidewire, switching stick, and halfpipe instruments with the shoulder in 20° of external rotation (Fig 5, Fig 6, Fig 7 and Video 1). The aiming guide has a marking at 3 cm, allowing the surgeon to decide the proportion of the subscapular tendon to be included in the sling.

**Fig 8:**
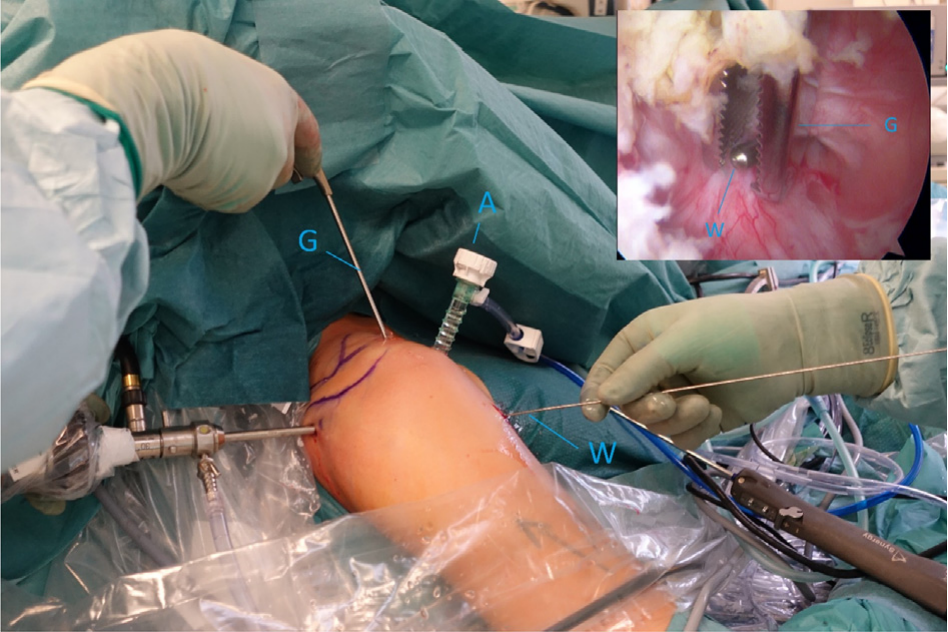
Right shoulder with a supplementary arthroscopic view, posterior viewing portal. Guidewire (W) is in the anterolateral portal, and the grasper (G) is in the anterosuperior portal to control right position of the guidewire.

**Fig 9:**
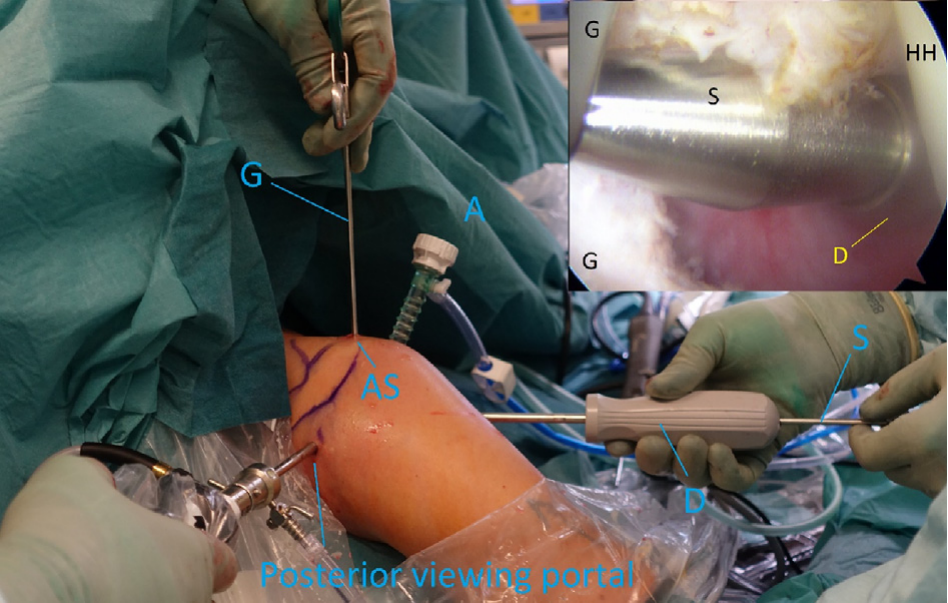
Right shoulder with a supplementary arthroscopic view, posterior viewing portal. Switching stick (S) and dilator (D) are in the anterolateral portal. (A, anterior portal; AS, anterosuperior portal; G, Grasper; HH, humeral head.)

**Fig 10:**
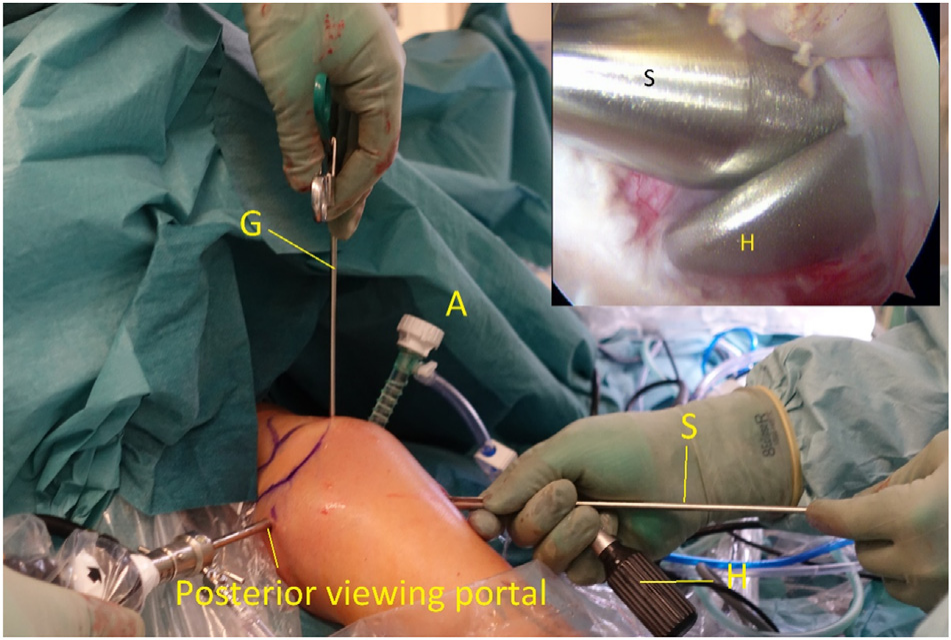
Right shoulder with a supplementary arthroscopic view, posterior viewing portal. Switching stick (S) and halfpipe (H) are in the anterolateral portal. Grasper (G) is in the anterosuperior portal and cannula in the anterior portal (A).

After the correct placement of the aiming guide, a long, cannulated needle is advanced into the joint and a switching stick slid carefully over it (Figs 8 and 9). Dilators of increasing size are advanced over the switching stick and finally, a halfpipe instrument is placed through the subscapular tendon into the joint (Figs 9 and 10).

**Fig 11:**
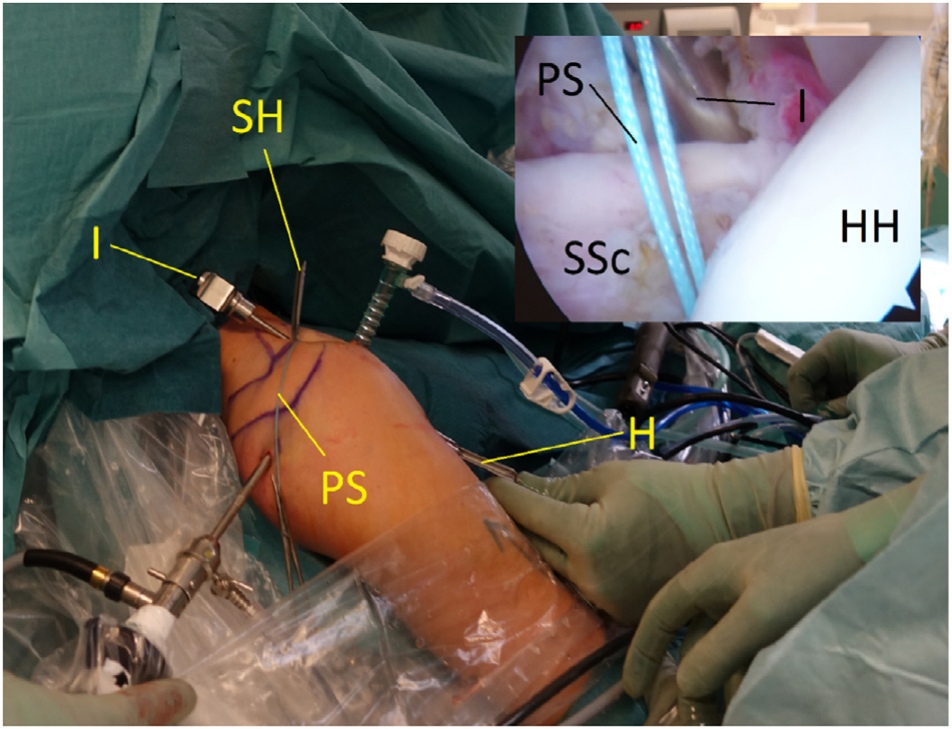
Right shoulder with a posterior arthroscopic view. Halfpipe (H) in the anterolateral portal, small halfpipe (SH) in the anterosuperior portal with the introducer (I). The pulling suture (PS) is situated posterior of the subscapular tendon (SSc) and the introducer (I) anterior of the subscapular tendon out through the anterolateral portal, humeral head (HH).

**Fig 12:**
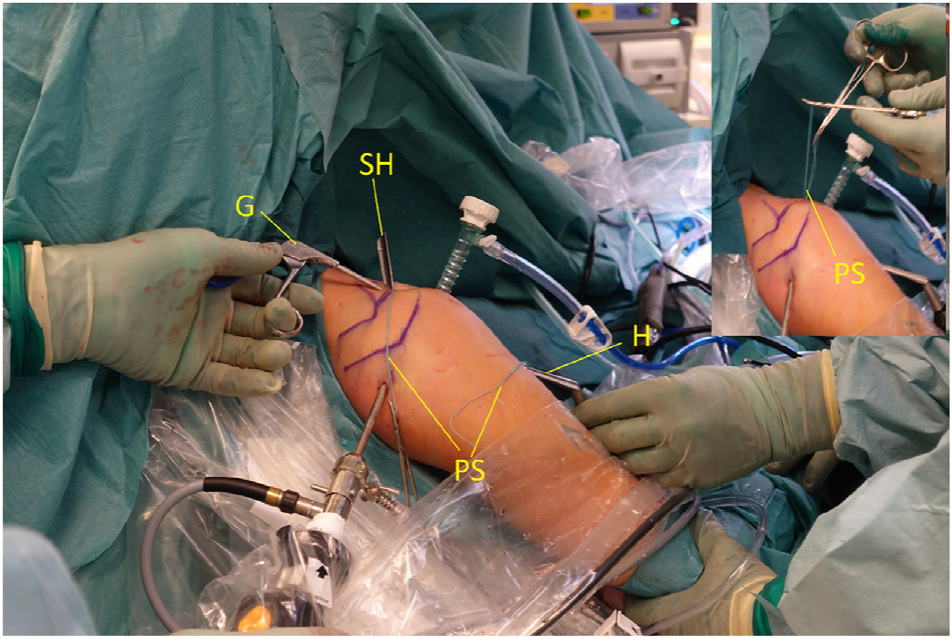
Right shoulder. The pulling suture (PS) with one limb in the anterosuperior portal and the other limb in the anterolateral portal. Small halfpipe (SH), halfpipe (H), and grasper (G) facilitate the placement of the pulling suture around the subscapular tendon with both limbs out of the anterosuperior portal (small picture). The correct placement of the pulling suture is verified before the graft is pulled into the joint.

**Fig 13:**
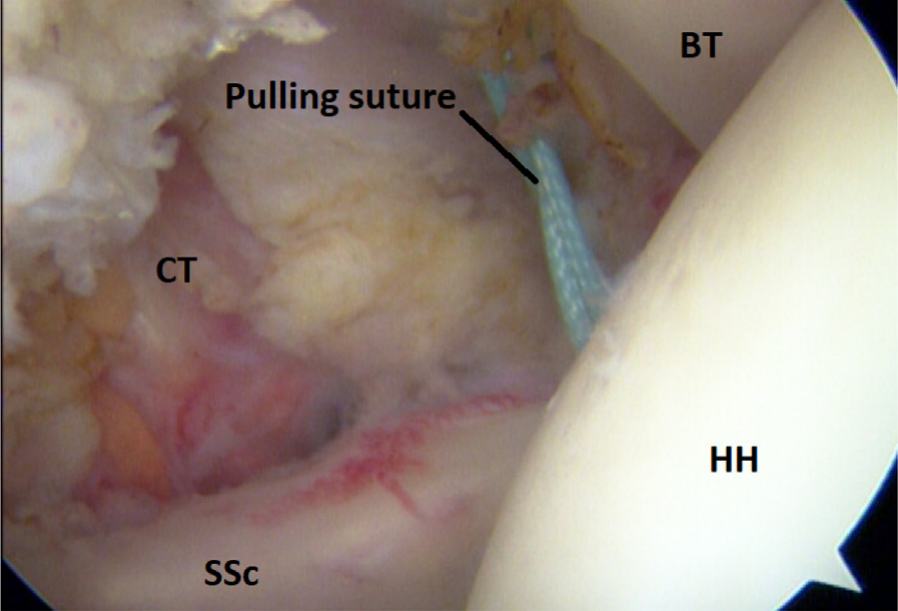
Arthroscopic view of a right shoulder, posterior viewing portal. Ti-cron #2 pulling suture situated posterior and anterior of the subscapular tendon and lateral of the conjoined tendon (CT). (BT, biceps tendon; HH, humeral head; SSc, subscapular tendon).

**Fig 14:**
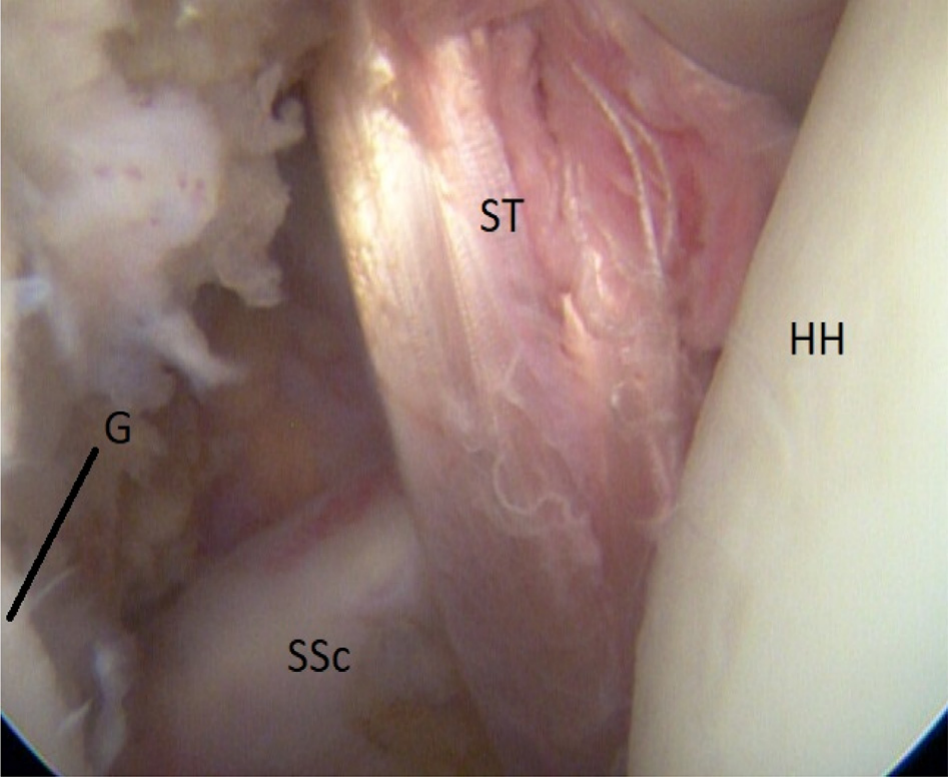
Intraoperative view of a right shoulder, posterior viewing portal. The semitendinosus graft (ST) is pulled into the joint and through the subscapular slit around the subscapular tendon (SSc). (G, anterior glenoid rim; HH, humeral head.)

A banana knife or a coblation device is passed over the halfpipe and used to make a slit in the longitudinal direction between the lower and middle third of the subscapular tendon. This is performed with a neutral rotation of the shoulder and cutting from medial to lateral under visual control. A short switching stick or blunt round instrument is advanced on the anterior wall of the subscapular tendon through the anterosuperior portal down to the halfpipe to facilitate the placement of a pulling suture (#2) and subsequently the ST-graft (Figs 6 and 11). With the help of a grasper, the pulling suture (#2) is introduced into the joint between the glenoid and the posterior wall of the subscapular tendon from the anterosuperior portal through the slit in the subscapular tendon and back out through the anterosuperior portal (Figs 6, 12, and 13). The tendinous graft end is connected to this suture and pulled into the joint and around the subscapular tendon through the anterosuperior portal (Fig 14).

**Fig 15:**
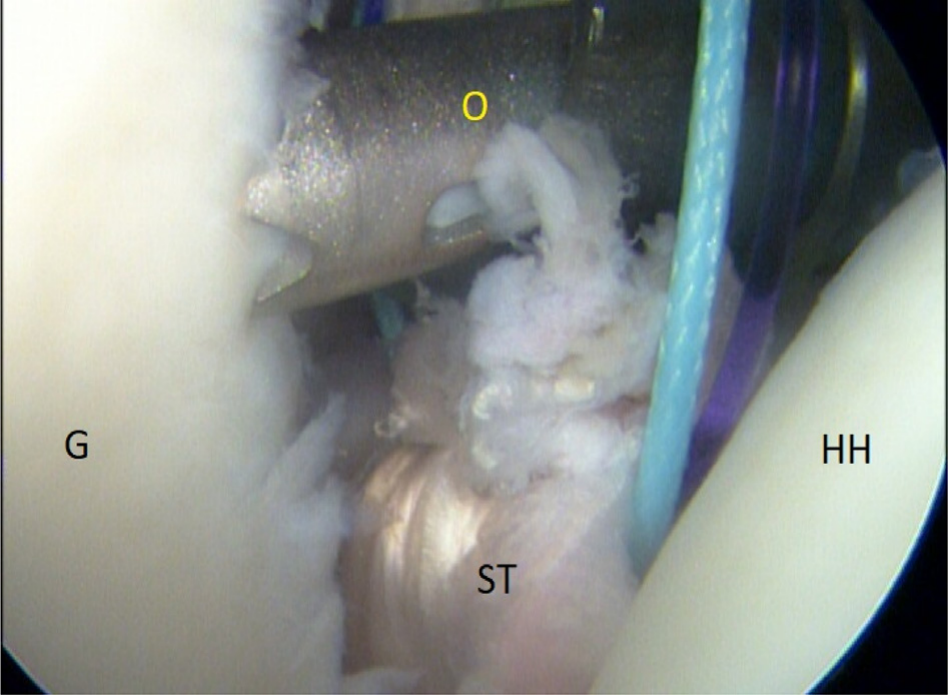
Arthroscopic view of a right shoulder, posterior viewing portal. Placement of the first anchor at 3 o’clock through the obturator (O). (G, glenoid; HH, humeral head; ST, semitendinosus graft.)

**Fig 16:**
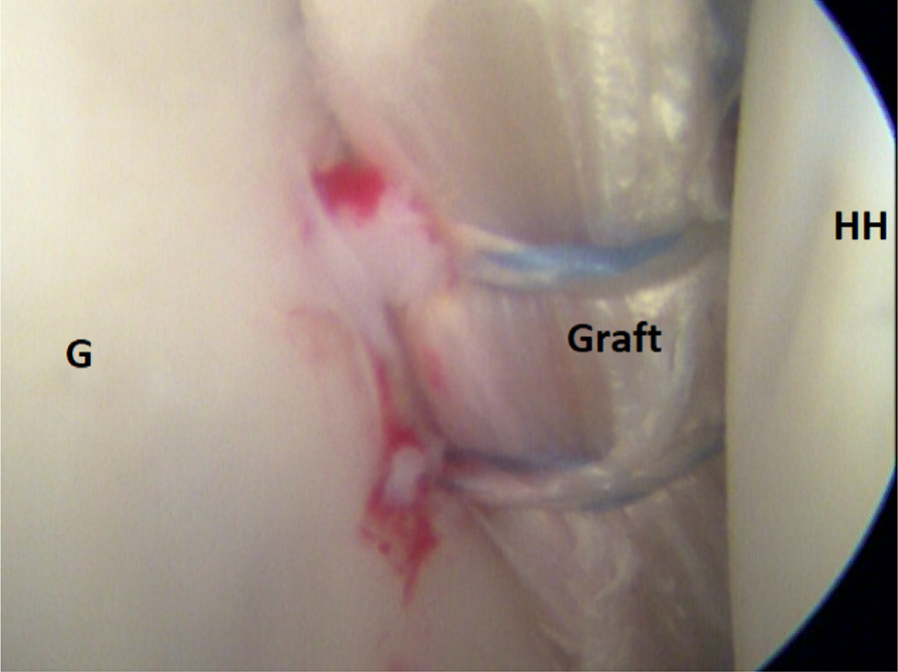
Arthroscopic view of a right shoulder, posterior viewing portal. The attached semitendinosus graft is on the anterior glenoid rim. (G, glenoid; GH, humeral head.)

**Fig 17:**
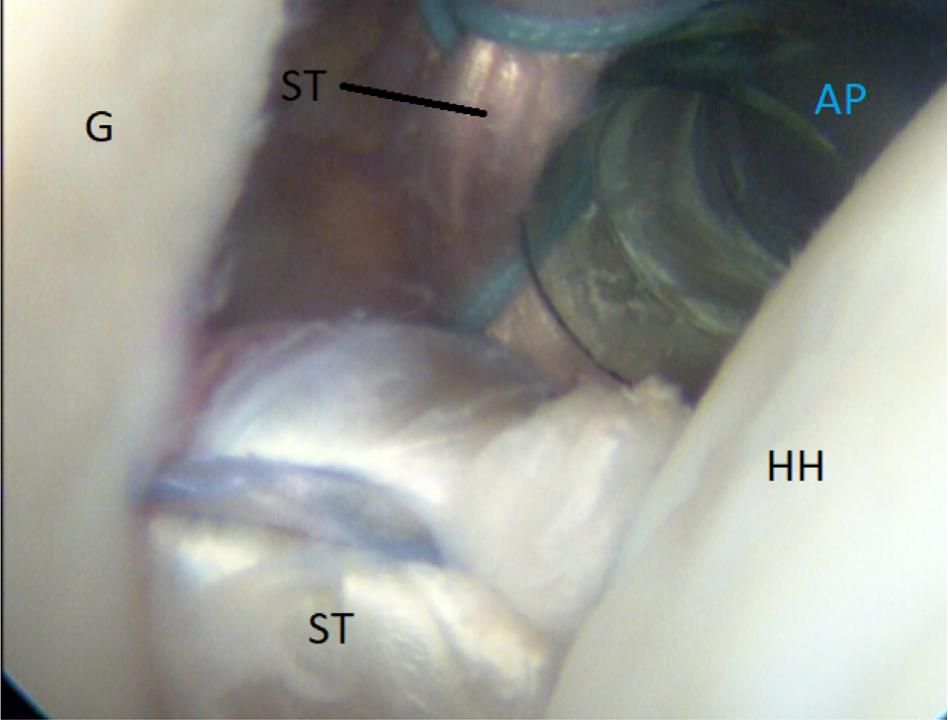
Right shoulder, posterior viewing portal. Arthroscopic view of the correct placement of semitendinosus (ST) graft on the anterior rim is shown. Medial of the anterior portal (AP) is the anterior limb of the ST graft before final fixation of the sling.

**Fig 18:**
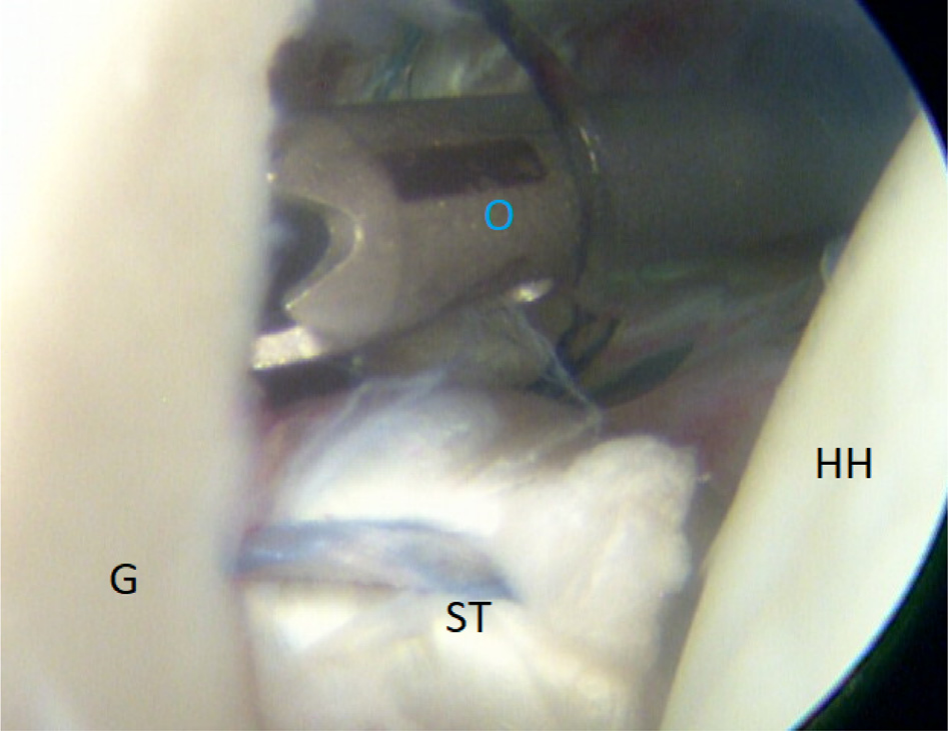
Arthroscopic view of a right shoulder, posterior viewing portal, is shown. Placement of an anchor at 2:30 through the obturator (O). (G, glenoid; HH, humeral head; ST, semitendinosus graft.)

**Fig 19:**
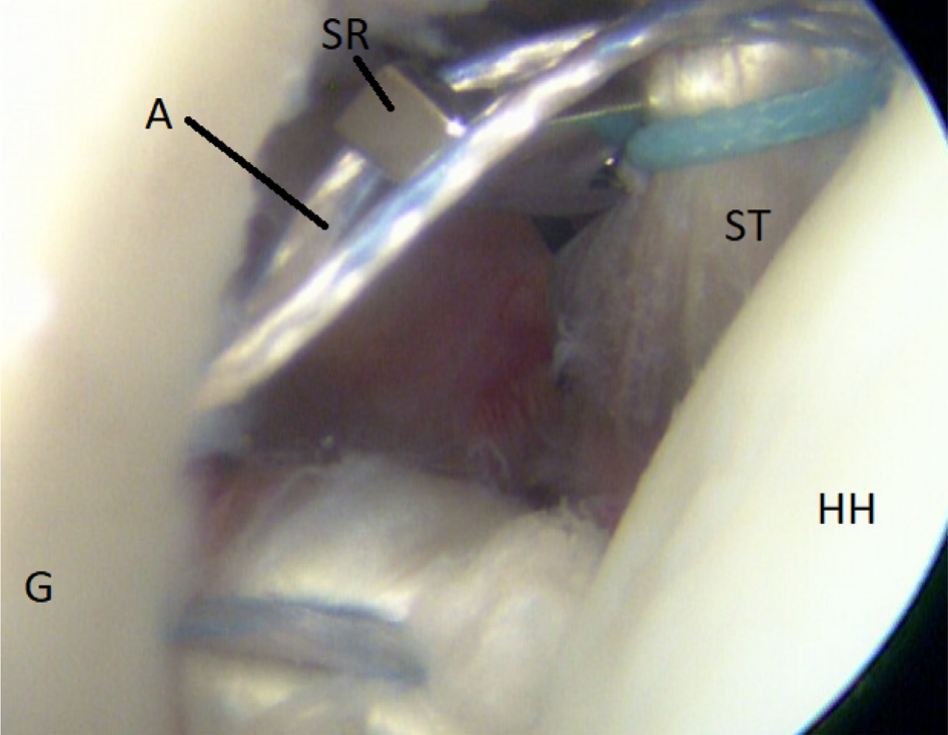
Arthroscopic view of a right shoulder, posterior viewing portal, is shown. Anchor at 2:30. Suture retriever (SR) pulls a strand from the anchor (A) on the medial side of the semitendinosus (ST) graft to achieve rigid fixation. (G, glenoid; HH, humeral head.)

**Fig 20:**
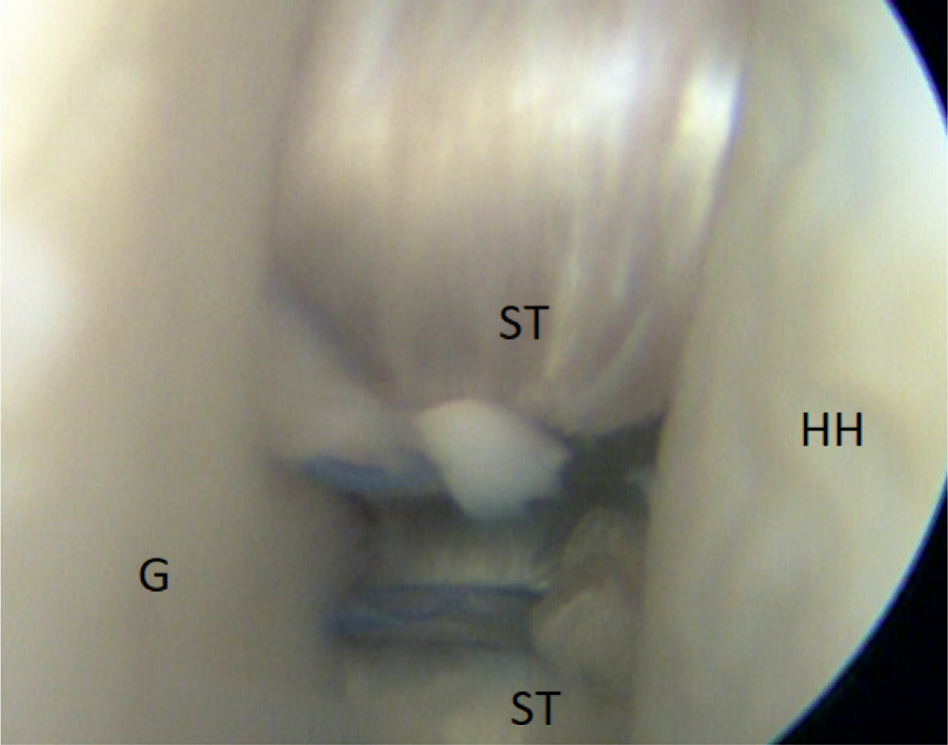
Final construct of the semitendinosus (ST) graft-sling on the anterior rim of the glenoid (G). Reconstruction of the lost anterior labrum is achieved. Humeral head (HH) is centered in the glenoid (G) cavity.

It is of immense importance to keep the shoulder in at least 20° of internal rotation at this point of the procedure to avoid impingement of the graft between the humeral head and the subscapular tendon. An external rotation would hinder the gliding and correct placement of the ST graft. FiberTak Soft anchors (Arthrex, Naples, FL) are placed from the 3- to 5-o’clock position to fixate the graft to the anterior glenoid rim to replace the lost anterior labrum (Figs 15 and 16). After the firm attachment is confirmed with a grasper, the remaining end of the graft, situated on the anterior side of the subscapular tendon, is pushed into the glenoid rim at approximately the 2 o’clock position. Two additional anchors are placed at this level, and the suture limbs are pulled through the ST-graft fibers to achieve a solid attachment (Fig 17, Fig 18, Fig 19, Fig 20).

To prevent a reduction in external rotation and abduction, the arm is at this point of the procedure held in 30° of abduction and 20° of external rotation to prevent the sling being too tight. The remaining graft is cut and removed from the joint. The graft fixation is assessed and the shoulder advanced carefully into the 90/90 position to judge the stability and containment of the joint. The joint is flushed, and the portals and camera are removed. The small incisions are sutured, and the shoulder is placed in a supportive sling for 4 weeks. Patients are recommended a postoperative rehabilitation program instructed by a physiotherapist. Pendulum and limited rotational exercises are started on the first postoperative day.

## Discussion

**Fig 21:**
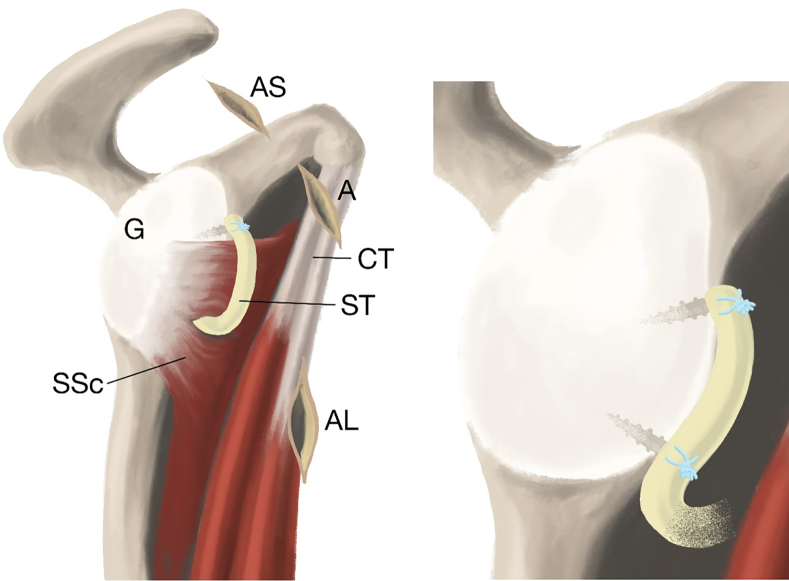
Schematic drawings of the completed sling. Attachment of the graft (ST) on the anterior glenoid (G) rim with anchors and the completed sling (ST) around the subscapular tendon (SSc). (A, anterior portal; AL, anterolateral portal; AS, anterosuperior portal; CT, conjoined tendon).

This paper presents the surgical technique of the novel subscapular ST sling. The sling supports the subscapular tendon and pulls it in a posterior direction to prevent an anterior dislocation. The ST graft replaces the lost anterior labrum and has, together with the subscapular tendon sling, both a static and dynamic stabilizing effect on the shoulder (Fig 21).

This procedure is an alternative option to other arthroscopic stabilizing procedures to treat anterior shoulder instability. After a Bankart stabilization, one third of the patients (mainly male patients younger than the age of 22 years) experience residual dislocation or do not return to their preinjury sporting level.^7^^,^^14^, ^15^, ^16^ In cases of previously performed soft-tissue stabilizing with recurrence, treatment options are usually different bone grafting procedures.^17^ The iliac crest bone grafting procedure and the Latarjet render good results but do have complications.^6^^,^^8^^,^^18^ The subscapular sling is part of a development in recent years seeking to find a surgical technique with fewer potential complications. Such a new procedure could fill the large gap between the Bankart fixation and the more difficult and anatomy-changing bone procedures. The pursuit of new procedures may also lead to the development of better arthroscopic instruments and subsequently improve the treatment options available.

Bone grafting procedures may change the anatomy of the shoulder. They are performed by both open surgery and arthroscopically.^3^^,^^19^ The proposed technique leaves the coracoid and adjacent tendons untouched and not transferred, which potentially reduces the risks of nerve injury, because portals and instrumentation are not needed medial to the conjoined tendon. A remplissage procedure could be advocated in engaging Hill–Sachs lesions,^20^^,^^21^ but has to our knowledge not been combined with the subscapular sling. Table 1 displays the advantages and disadvantages of the subscapular sling procedure.

**Fig 22:**
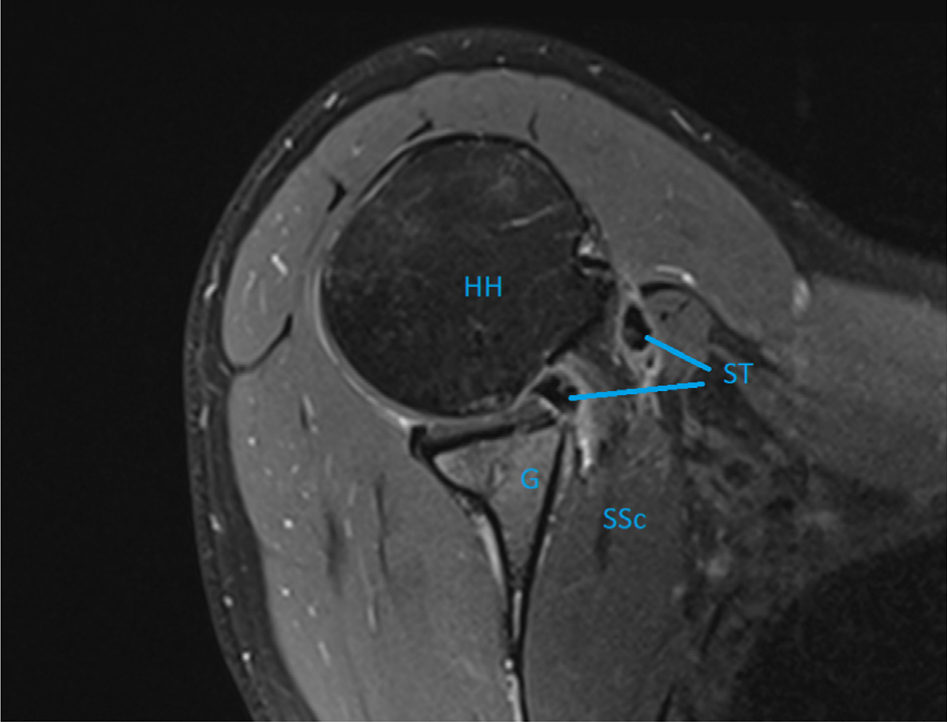
Sagittal magnetic resonance imaging of a right shoulder displaying the intact semitendinosus sling (ST) and the subscapular tendon/muscle (SSc). (G, glenoid; HH, humeral head.)

The drawbacks of this procedure include the use of an ST autograft, but allografts may also be an option. There are reports of reduction in flexion strength at the donor site, but sequalae are otherwise limited to postoperative pain and loss of sensation around the donor site.^22^^,^^23^ Patients scheduled for this operation must be informed about the potential of pain, thrombosis, and loss of sensation. Postoperative administration of low-molecular-weight heparin should be considered to avoid thrombosis. The long-term effects of this technique are unknown. Postoperative magnetic resonance imaging has confirmed an intact reconstructed labrum, sling, and subscapular tendon (Fig 22). The sling may nevertheless cause erosion and rupture of the subscapular tendon. Ruptures of such kind or muscle atrophy of the subscapularis have not been detected.

The graft-sling and the subscapular tendon can eventually lose firmness and strength and subsequently reduce the stability of the shoulder. Firm attachment and ingrowth of the graft on the anterior glenoid rim are essential to achieve a negative joint pressure and suction effect. These effects may diminish if the healing is disturbed by poor graft quality and/or bone quality. The tightness of the sling is important to assess during surgery. An overconstrained sling would hinder the external rotation and may also, in the long run, induce arthritis because of the excessive pressure on the anterior part of the glenohumeral joint.

Table 2 displays the indications and contraindications for this procedure. A torn subscapular tendon is an absolute contraindication because the dynamic stability of the subscapular sling is dependent on an unimpaired tendon. An elongated subscapular tendon with a partial tear may be a relative contraindication. The subscapular sling has not been tested with substantial glenoid bone loss and should in these cases be avoided until further studies have investigated its efficacy. A further development could be to use a quadriceps tendon–bone graft in shoulders with severe glenoid bone loss.^24^ The subscapular sling procedure with a ST graft may in future be included in the possible treatment options for patients suffering from residual anterior shoulder instability.

**Table 1:** Advantages and Disadvantages of the Subscapular Sling Procedure

Advantages	Disadvantages
Arthroscopic technique—easier learning curve	No extension of the glenoid bone margins
Less invasive and minimal change of anatomy	Graft is smaller than the conjoined tendon
Portals lateral of conjoined tendon with less risk of nerve injury	Dependent on full integrity of the subscapular tendon
No overhang of bone on the anterior rim	Semitendinosus autograft with possible sequalae
Soft-tissue reconstruction of labrum with the graft	
No tenodesis of subscapular tendon

**Table 2:** Suggested Indications and Contraindications for the Subscapular Sling Procedure

Indications	Contraindications
Residual anterior shoulder instability	Posterior and multidirectional instability
Intact subscapular tendon	Torn subscapular tendon
Previous Bankart repair	Previous bony procedures
Minimal glenoid bone loss	Severe glenoid bone loss

## Supplementary Data

Video 1 The subscapular sling procedure. This procedure uses a semitendinosus graft as a sling around the upper two-thirds of the subscapular tendon, attached to the anterior glenoid rim. The reconstruction of the labrum and the sling provide both static and dynamic stabilization of the shoulder.

ICMJE author disclosure forms
